# Aminoglycoside 6′-*N*-acetyltransferase Type Ib [AAC(6′)-Ib]-Mediated Aminoglycoside Resistance: Phenotypic Conversion to Susceptibility by Silver Ions

**DOI:** 10.3390/antibiotics10010029

**Published:** 2020-12-31

**Authors:** Craig M. Reeves, Jesus Magallon, Kenneth Rocha, Tung Tran, Kimberly Phan, Peter Vu, Yang Yi, Crista L. Oakley-Havens, José Cedano, Verónica Jimenez, Maria S. Ramirez, Marcelo E. Tolmasky

**Affiliations:** Center for Applied Biotechnology Studies, Department of Biological Science, California State University Fullerton, Fullerton, CA 92831, USA; wizard5424@csu.fullerton.edu (C.M.R.); jesusmagallon91@csu.fullerton.edu (J.M.); kenneth.rocha@csu.fullerton.edu (K.R.); tungtran6186@yahoo.com (T.T.); kkphan@csu.fullerton.edu (K.P.); vupeter8@csu.fullerton.edu (P.V.); yiyang6655@csu.fullerton.edu (Y.Y.); cristalee0810@csu.fullerton.edu (C.L.O.-H.); Jcedano@csu.fullerton.edu (J.C.); vjimenezortiz@Exchange.FULLERTON.EDU (V.J.); msramirez@fullerton.edu (M.S.R.)

**Keywords:** ESKAPE, *Acinetobacter*, aminoglycosides, amikacin, acetyltransferase, silver, adjuvant

## Abstract

Clinical resistance to amikacin and other aminoglycosides is usually due to the enzymatic acetylation of the antimicrobial molecule. A ubiquitous resistance enzyme among Gram-negatives is the aminoglycoside 6′-*N*-acetyltransferase type Ib [AAC(6′)-Ib], which catalyzes acetylation using acetyl-CoA as a donor substrate. Therapies that combine the antibiotic and an inhibitor of the inactivation reaction could be an alternative to treat infections caused by resistant bacteria. We previously observed that metal ions such as Zn^2+^ or Cu^2+^ in complex with ionophores interfere with the AAC(6′)-Ib-mediated inactivation of aminoglycosides and reduced resistance to susceptibility levels. Ag^1+^ recently attracted attention as a potentiator of aminoglycosides′ action by mechanisms still in discussion. We found that silver acetate is also a robust inhibitor of the enzymatic acetylation mediated by AAC(6′)-Ib in vitro. This action seems to be independent of other mechanisms, like increased production of reactive oxygen species and enhanced membrane permeability, proposed to explain the potentiation of the antibiotic effect by silver ions. The addition of this compound to *aac(6′)-Ib* harboring *Acinetobacter baumannii* and *Escherichia coli* cultures resulted in a dramatic reduction of the resistance levels. Time-kill assays showed that the combination of silver acetate and amikacin was bactericidal and exhibited low cytotoxicity to HEK293 cells.

## 1. Introduction

Silver has been used for the treatment of human diseases since ancient times [1,2]. In particular, until the advent of antibiotics in the mid-20th century, silver may have been the most used medicine to treat infections [2]. Despite taking a backstage role after the advent of antibiotics, silver has been continuously used in different forms [1,2]. Furthermore, due to the multidrug resistance crisis, the utilization of silver as a component of combination therapies or other novel formulations has regained interest [3,4,5,6,7].

Multidrug resistant Gram-negatives are one of the most serious threats to human health [8,9]. In particular, *Acinetobacter baumannii* is responsible for a large fraction of multiresistant hospital outbreaks [10,11]. Infections caused by this bacterium present multiple clinical manifestations, high mortality, and refraction to treatment [12,13,14]. These characteristics positioned *A. baumannii* within the U.S. Centers for Disease Control′s list of threats to human health [15,16]. Amikacin and other aminoglycosides are important components of the armamentarium against *A. baumannii* and other bacterial infections [17,18]. Furthermore, based on recent docking experiments, amikacin could be useful in the treatment of viral infections including COVID-19 [19]. Unfortunately, a substantial percentage of *A. baumannii* clinical isolates have acquired resistance to these antibiotics. A common mechanism of resistance to aminoglycosides is the enzymatic transfer of an acetyl group from acetyl-CoA to the 6′ amine group of the antibiotic molecule. The aminoglycoside 6′-*N*-acetyltransferase type Ib [AAC(6′)-Ib], an enzyme coded for by the *aac(6′)-Ib* gene found in integrons, transposons, plasmids, and chromosomes of Gram-negative bacteria, is responsible for most amikacin-resistant strains [20,21]. A strategy to regain aminoglycosides′ effectiveness and reduce the risks of infections caused by multidrug resistant Gram-negatives is to develop inhibitors of the enzymatic reaction that could be administered in combination with the antibiotic [20,22,23]. This path proved to be successful in the case of β-lactamase-mediated resistance to β-lactams [22,24]. The recent finding that metal ions such as Zn^2+^ and Cu^2+^, in complex with ionophores, inhibit the acetylation of aminoglycosides mediated by AAC(6′)-Ib and reverse amikacin resistance in laboratory assays increased the expectations that viable formulations that can treat infections caused by resistant *A. baumannii* will be designed in the near future [25,26,27,28]. In this article, we describe the inhibition of AAC(6′)-Ib-mediated amikacin-resistance by Ag^1+^ in *A. baumannii* and *E. coli*.

## 2. Results

### 2.1. Effect of Ag^1+^ on AAC(6′)-Ib-Mediated Acetylation of Amikacin

**Figure 1:**
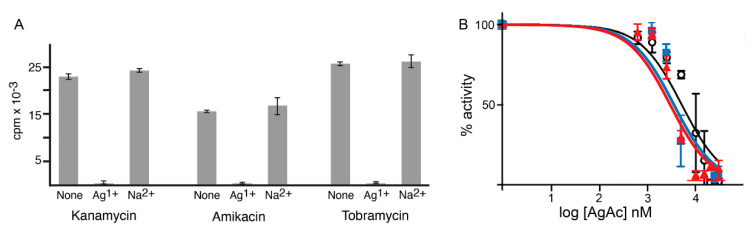
Effect of Ag^1+^ on AAC(6′)-Ib activity. (**A**) acetylating activity of AAC(6′)-Ib using kanamycin, tobramycin, or amikacin as substrates in the presence of Ag^1+^. Silver acetate (AgAc) and sodium acetate were added at 100 μM and the activity was compared to that observed in its absence. (**B**) The percentage of acetylating activity by AAC(6′)-Ib was calculated for reaction mixtures containing different concentrations of silver acetate.

Ag^1+^ drastically interfered with the acetylation of amikacin, kanamycin, and tobramycin catalyzed by AAC(6′)-Ib. Figure 1A shows that while the addition of sodium acetate did not produce any changes in the acetylation levels, silver acetate completely obliterated the incorporation of an acetyl group to the aminoglycoside molecule. The strength of inhibition was assessed by determining the 50% inhibitory concentration values (IC50) using kanamycin, tobramycin, or amikacin as substrates. The values found were 5.1, 3.5, and 3.1 μM, respectively (Figure 1B).

### 2.2. Silver Acetate Interferes with AAC(6′)-Ib-Mediated Resistance to Amikacin

**Figure 2:**
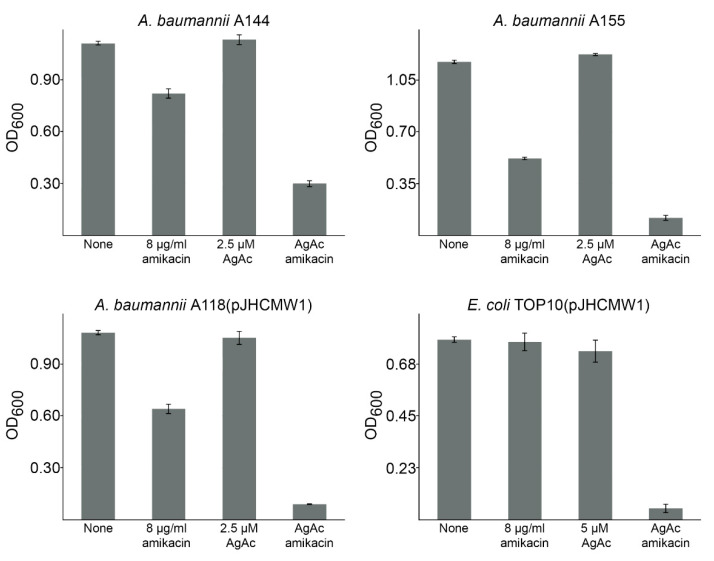
Effect of Ag^1+^ on AAC(6′)-Ib-mediated resistance to amikacin. *A*. *baumannii* A155, A144, and A118(pJHCMW1), and *E. coli* TOP10(pJHCMW1) were cultured in 100 μL Mueller–Hinton broth in microtiter plates at 37 °C, with the additions indicated in the figure and the OD_600_ was determined after 20 h. The concentrations when both compounds were added to the cultures are those used when they were added as single addition. *p* values of the cultures containing silver acetate (AgAc), and amikacin were calculated with respect to the results obtained in cultures containing only amikacin. All four *p* values were statistically significant (<0.05).

To determine if the silver acetate-mediated inhibition of AAC(6′)-Ib activity observed in vitro has a significant impact on bacterial resistance, we assessed the effect on the growth of *aac(6′)-Ib*-harboring *A. baumannii* and *E. coli* strains in amikacin containing media. Figure 2 shows that silver acetate dramatically reduced the growth of all these otherwise amikacin-resistant strains. Cultures in the presence of silver acetate were not affected at all, while partial inhibition of growth was observed in the presence of amikacin. These results showed that the presence of Ag^1+^ interferes with resistance to amikacin mediated by AAC(6′)-Ib.

### 2.3. Bactericidal Effect

**Figure 3:**
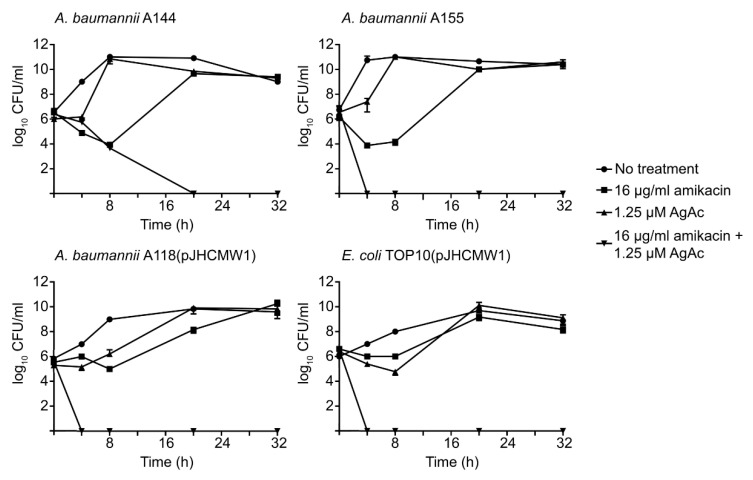
Time-kill assay curves for amikacin in the presence of silver acetate. *A*. *baumannii* A155, A144, and A118(pJHCMW1), and *E. coli* TOP10(pJHCMW1) cultures in Mueller-Hinton broth were incubated until they contained the indicated CFU/mL. Then, the different compounds were added, and the cultures were incubated at 37 °C. The CFU/mL values were measured at different intervals. AgAc, silver acetate.

We carried out time-kill assays to confirm that the severe reduction of growth observed when all four strains were cultured in the presence of the combination of silver acetate and amikacin was due to a bactericidal effect. Figure 3 shows that in tests using *A*. *baumannii* A144, A155, A118(pJHCMW1), and *E. coli* TOP10(pJHCMW1), the addition of silver acetate and amikacin had a robust bactericidal effect. As expected, these strains showed healthy growth when one of the components of the mix was omitted. These results confirmed that amikacin in the presence of Ag^1+^ ions regained full bactericidal power when resistance was mediated by the AAC(6′)-Ib enzyme.

### 2.4. Cytotoxicity

**Figure 4:**
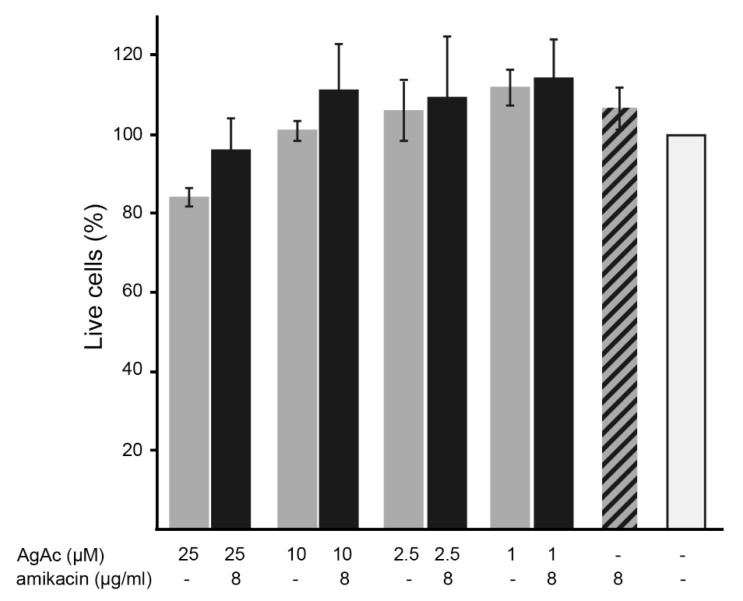
Cytotoxicity of silver acetate (AgAc) and amikacin. Cytotoxicity of silver acetate alone and in combination with amikacin on HEK293 cells was assayed using a LIVE/DEAD kit. The percentage of surviving cells was calculated relative to cells untreated (white bar). Cells incubated with 70% methanol were used as a control of maximum toxicity (striped bar). The experiments were conducted in triplicate and the values are mean ± SD.

The cytotoxicity of the mix of silver acetate and amikacin, as well as that of the individual components, were tested on HEK293 cells as described in the Materials and Methods section. Addition of silver acetate or amikacin alone or in combination at the concentrations required to overcome resistance did not cause significant mortality in treated cells with respect to the control (Figure 4).

## 3. Discussion

The antibiotic resistance crisis has triggered the interest in finding creative alternatives to extend the life of antibiotics currently in use. Since one of the most ubiquitous acquired mechanisms of resistance to aminoglycosides is their enzymatic modification, compounds that interfere with this process would fulfill the purpose stated above. Numerous compounds have been proposed as candidates to inhibit enzymatic modification of aminoglycosides [20,22,23,29,30,31]. In the case of the AAC(6′)-Ib, the most common aminoglycoside resistance enzyme among AAC(6′)-I-carrying Gram-negatives [21,32], some small molecules have been identified that act as enzymatic inhibitors [20,23,29,33,34,35]. In addition to these advances, recent research revealed that Zn^2+^ and other metal ions inhibit the acetylation of aminoglycosides mediated by AAC(6′)-Ib in vitro [25,26,27,28,36,37]. The mechanism by which these metal ions interfere with enzymatic acetylation of aminoglycosides mediated by AAC(6′)-Ib is not yet known. A hypothesis has been put forward proposing the formation of a coordination complex that protects the substrate aminoglycoside from modification [26,38]. This manuscript shows that silver ions are also potent inhibitors of the enzymatic inactivation of aminoglycosides. However, while to overcome resistance to aminoglycosides in the presence of Zn^2+^ or Cu^2+^ ions at low concentrations, they had to be complexed to ionophores like pyrithione or clioquinol [25,26,27,28], the addition of low μM concentrations of silver acetate was sufficient. Our experiments showed that concentrations lower than 10 μM of silver acetate completely abolish resistance to amikacin in *aac(6′)-Ib*-carrying *A. baumannii* and *E. coli* strains. Furthermore, time-kill assays confirmed that the effect of the combination amikacin/silver acetate was bactericidal.

The potentiating effect of Ag^1+^ ions on aminoglycoside antibiotics has been recently reported [4,7]. One of these reports proposed that an increase in reactive oxygen species production is the primary mechanism by which silver ions act as adjuvants [4]. On the other hand, another series of experiments pointed to an enhanced uptake level of antibiotics induced by silver ions as the molecular mechanism behind the observed activity [7]. Furthermore, the authors found no correlation between the silver activity and levels of reactive oxygen species [7]. While in these studies all experiments were carried on growing bacterial cells, our assays included the demonstration of interferences with acetylation in in vitro enzymatic reactions. Our results do not discard the possibilities proposed by the earlier work [4,7] but positively add another mechanism of enhancing resistance to aminoglycosides by inhibiting the enzymatic modification catalyzed by AAC(6′)-Ib. The results reported by other researchers [4,7] along with those described in this article led us to conclude that silver could act in vivo at multiple levels resulting in a synergistic effect that obliterates the resistance. The increase in membrane permeability produced by Ag^1+^ [4,7] may be why an ionophore was not necessary to keep the active concentrations of the adjuvant at the μM levels. The mechanism by which silver increases permeability might be a combination of disturbances, including direct alteration of membrane proteins or an effect of the metal on ribosomes that interferes with proper translation and results in aborted and misfolded membrane proteins [1,4,7].

Silver has been used to treat infections since ancient times; the discovery that it can also act as potentiator of antimicrobials, in particular aminoglycosides, by numerous mechanisms that include the inhibition of aminoglycoside modifying enzymes, which can be very active and exist in high concentrations in the cytoplasm of resistant strains, makes combinations between Ag^1+^ and aminoglycosides excellent candidates to treat multidrug resistant infections. The excitement about the possibility of using these mixes as therapeutic agents for a variety of infections is enhanced by the low toxicity the mix exhibited in our tests.

## 4. Materials and Methods

### 4.1. Bacterial Strains and Plasmids

Plasmids and strains used in this work are described in Table 1. All three *A. baumannii* strains used in this study are clinical isolates. Two of them, A144 and A155, naturally carry *aac(6′)-Ib*. *A. baumannii* and *E. coli* TOP10 were transformed with the plasmid pJHCMW1, which harbors *aac(6′)-Ib*.

### 4.2. General Procedures

Bacteria were cultured in Lennox L broth (1% tryptone, 0.5% yeast extract, 0.5% NaCl), and 2% agar was added in the case of solid medium. Inhibition of *A*. *baumannii* strains growth was determined in Mueller-Hinton broth at 37 °C with shaking in microtiter plates. The optical density at 600 nm (OD_600_) of the cultures containing the specified additions was determined after 20 h incubation at 37 °C using the BioTek Synergy 5 microplate reader as described before [27]. Amikacin sulfate concentration is expressed in the usual μg/mL units; these numbers multiplied by 1.27 give the μM concentration. For time-kill assays, the cells were cultured in Mueller-Hinton broth until they reached 10^6^ CFU/mL before adding the indicated compounds. The cultures were continued at 37 °C with shaking, and the number of cells was determined by taking aliquots after 0, 4, 8, 20, and 32 h [28]. *E*. *coli* TOP10 and *A*. *baumannii* A118 were transformed with pJHCMW1 DNA [44] as described by Cohen et al. [45] and Ramirez et al. [46], respectively. Plasmid DNA preparations and DNA gel extractions were performed with the QIAprep Spin miniprep kit and QIAquick gel extraction kit, respectively (QIAGEN). Purification of AAC(6′)-Ib was carried out as before [29].

### 4.3. Acetyltransferase Assays

Acetyltransferase activity was assessed using two methodologies. One of them, the phosphocellulose paper binding assay [47], was carried out as previously described [48,49]. Briefly, 120 μg of protein from a soluble extract obtained from sonically disrupted *E. coli* TOP10(pJHCMW1) cells were added to a reaction mixture containing 200 mM Tris HCl pH 7.6 buffer, 0.25 mM MgCl_2_, 330 μM antibiotic, 100 μM sodium acetate or silver acetate, and 0.05 μCi of [acetyl-1-^14^C]-acetyl-coenzyme A (specific activity 60 μCi/μmol) in a final volume of 30 μL. There mixture was incubated at 37 °C for 15 min and 20 μL were spotted on phosphocellulose paper strips. The unreacted radioactive substrate was washed once by submersion in 80 °C water followed by two washes with room temperature water. After drying, the radioactivity corresponding to the acetylated antibiotic was determined. The other method, utilized to calculate IC_50_ values, consisted of monitoring the increase in absorbance at 412 nm when the released CoA-SH from the substrate acetyl-CoA reacts with Ellman′s reagent [5,5′-dithiobis(2-nitrobenzoic acid, DTNB)] [50]. The reaction mixture (150 μM acetyl CoA, 0.2 mM DTNB, 20 mM Tris-HCl pH 7.5 buffer, 18 μM antibiotic, and the required concentrations of silver acetate) was incubated at room temperature for 10 min. At this moment purified AAC(6′)-Ib was added and acetylation was followed using a BioTek Synergy 2 plate reader monitoring absorbance at 412 nm. The initial velocities were calculated using the Gen 5 software, version 2.01.13. Inhibition was assessed by comparison of initial velocities of acetylation reactions in the presence or absence of silver acetate.

### 4.4. Cytotoxicity Assays

The cytotoxicity of the formulation silver acetate/amikacin was assessed on HEK293 cells [51] as described previously [29]. Briefly, 10^3^ cells per well were inoculated and cultured overnight using flat-bottom 96-well, black microtiter plates. After this period, the compounds to be tested were added to cell-containing wells at the concentrations indicated, and incubation was continued for 24 h. At this point, the cells were washed with sterile D-PBS, resuspended in the LIVE/DEAD reagent (2 μM ethidium homodimer 1 and 1 μM calcein-AM) (Molecular Probes), and incubated for 30 min at 37 °C before determining the fluorescence levels at 645 nm (dead cells) and 530 nm (live cells). The percentage of dead cells was calculated relative to the untreated control cells. The maximum toxicity control was calculated treating the cells with 70% methanol for 10 min. Experiments were conducted in triplicate. The results were expressed as mean ± SD of three independent experiments.

## 5. Conclusions

An enhancing effect of Ag^1+^ ions on aminoglycosides had been recently reported. The causes proposed by different research groups were an increase in the production of reactive oxygen species and an enhanced uptake level of antibiotics induced by silver ions. The results showed here indicate that besides these mechanisms, Ag^1+^ inhibits the acetylation of aminoglycosides catalyzed by AAC(6′)-Ib. Addition of silver acetate at concentrations lower than 10 μM sufficed to dramatically reduce the resistance to amikacin in *A. baumannii* and *E. coli* strains carrying the *aac(6′)-Ib* gene. This observation, together with the low toxicity, make combinations of amikacin and silver acetate excellent candidates to treat infections caused by multidrug resistant Gram-negatives.

## Figures and Tables

**Table 1:** Bacterial strains and plasmids used in this study.

Bacterial Strain	Relevant Characteristics, Genotype or Phenotype	Source or Reference
*E. coli*		
TOP10(pJHCMW1)	TOP10 (F^-^ *mcrA* Δ(*mrr-hsd*RMS-*mcr*BC) Φ80*lac*ZΔM15 Δ*lac*X74 *rec*A1 *ara*D139 Δ(*ara-leu*)7697 *gal*U *gal*K *rps*L(Str^R^) *end*A1 *nup*G). Transformed with pJHCMW1	[39]
*A. baumannii*		
A144	Human clinical isolate. Naturally carries *aac(6′)-Ib*	[40]
A155	Human clinical isolate. Naturally carries *aac(6′)-Ib*	[41]
A118(pJHCMW1)	Human clinical isolate transformed with pJHCMW1	[27]
Plasmids		
pJHCMW1	A 17 copies/cell plasmid harboring *aac(6′)-Ib*	[42,43]

## Data Availability

Data is contained within the article.
